# Following Up Nonrespondents to an Online Weight Management Intervention: Randomized Trial Comparing Mail versus Telephone

**DOI:** 10.2196/jmir.9.2.e16

**Published:** 2007-06-13

**Authors:** Mick P Couper, Andy Peytchev, Victor J Strecher, Kendra Rothert, Julia Anderson

**Affiliations:** ^5^Group Health CooperativeSeattleWAUSA; ^4^Kaiser Permanente Care Management InstituteOaklandCAUSA; ^3^University of Michigan Center for Health Communications Research and HealthMediaIncAnn ArborMIUSA; ^2^RTI InternationalResearch Triangle ParkNCUSA; ^1^Institute for Social ResearchUniversity of MichiganAnn ArborMIUSA

**Keywords:** Nonresponse, attrition, Internet, weight management, randomized controlled trial

## Abstract

**Background:**

Attrition, or dropout, is a problem faced by many online health interventions, potentially threatening the inferential value of online randomized controlled trials.

**Objective:**

In the context of a randomized controlled trial of an online weight management intervention, where 85% of the baseline participants were lost to follow-up at the 12-month measurement, the objective was to examine the effect of nonresponse on key outcomes and explore ways to reduce attrition in follow-up surveys.

**Methods:**

A sample of 700 nonrespondents to the 12-month online follow-up survey was randomly assigned to a mail or telephone nonresponse follow-up survey. We examined response rates in the two groups, costs of follow-up, reasons for nonresponse, and mode effects. We ran several logistic regression models, predicting response or nonresponse to the 12-month online survey as well as predicting response or nonresponse to the follow-up survey.

**Results:**

We analyzed 210 follow-up respondents in the mail and 170 in the telephone group. Response rates of 59% and 55% were obtained for the telephone and mail nonresponse follow-up surveys, respectively.  A total of 197 respondents (51.8%) gave reasons related to technical issues or email as a means of communication, with older people more likely to give technical reasons for noncompletion; 144 (37.9%) gave reasons related to the intervention or the survey itself. Mail follow-up was substantially cheaper: We estimate that the telephone survey cost about US $34 per sampled case, compared to US $15 for the mail survey. The telephone responses were subject to possible social desirability effects, with the telephone respondents reporting significantly greater weight loss than the mail respondents. The respondents to the nonresponse follow-up did not differ significantly from the 12-month online respondents on key outcome variables.

**Conclusions:**

Mail is an effective way to reduce attrition to online surveys, while telephone follow-up might lead to overestimating the weight loss for both the treatment and control groups. Nonresponse bias does not appear to be a significant factor in the conclusions drawn from the randomized controlled trial.

## Introduction

Online interventions are an increasingly attractive method of reaching large numbers of potential participants for a wide variety of health interventions [1]. However, a key challenge of online health interventions is that of retaining subjects, especially for follow-up surveys to measure outcomes [2]. High rates of attrition, or dropout, can seriously threaten the inference from evaluations of online interventions, both in terms of external validity (generalizability) and internal validity [2, 3]. Online interventions appear to be particularly susceptible to problems of attrition. For example, only 1% of participants completed all 12 weeks of a panic disorder program [4], 33% completed all five modules of a depression program [5], and 35% completed a follow-up questionnaire about 10 weeks after enrollment in a smoking-cessation trial [6]. In his review of the problem, Eysenbach [2] argues for a “science of attrition,” saying that “Nonusage data *per se* should be of great interest to researchers, and attrition curves may be underreported and underanalyzed.”

While mixed-mode designs are increasingly common in survey research (for a review, see [7]), both as a way to reduce costs and to increase response rates, such mode switches are less common in health interventions, especially those involving online methods. In one exception, Tomson et al [8] reported on a telephone interview with nonresponders to a mail follow-up survey in a smoking intervention. Of the 84 subjects they followed up, 46 responded (for a 55% response rate). They found that those who did not respond to the original survey were more likely to be smoke-free at 12 months (39%) than the original mail respondents (31%). When asked about reasons for not returning the mail questionnaire, 35% claimed that they had returned it and 20%, that they had not received it.

While there are several comparisons of mail versus email for health surveys (eg, [9-12]), we are aware of no studies that have followed up nonrespondents to an online survey using an alternative mode of data collection. Crawford and colleagues [13] used telephone follow-up for nonrespondents to a mail and Web survey to remind people to complete the survey and to ascertain reasons for nonresponse, but not to collect follow-up data. Clarke and colleagues [14] assigned participants to a telephone or mail reminder, but similarly did not use these modes to collect follow-up data.

This paper represents an attempt to better understand the attrition problem in a randomized controlled trial (RCT) of an online weight management intervention and to find ways to counter the potential negative effect of attrition on the conclusions that can be drawn from such studies. We describe a follow-up procedure to examine why people drop out and what can be done about it. We explore alternative modes (mail and telephone) for following up nonresponders to the online surveys. Finally, we discuss the implications of this work for online interventions and follow-up surveys.

## Methods

### Background on the Online Intervention

This nonresponse follow-up study is part of a broader project aimed at evaluating tailored versus nontailored Web-based weight management materials. Details of the study have been described elsewhere [15]. Kaiser Permanente (KP) members in four regions of the United States were recruited using a variety of methods (through clinicians, member newsletters, and letters to members of diabetes and cardiovascular disease registries). A total of 4041 eligible participants were enrolled over a 6-month period beginning in September 2002. Eligible participants were current members of KP who were age 18 and older, had regular access to the Web and a functioning email address, had a body mass index (BMI) of 25 or greater, and who expressed a willingness to complete follow-up questionnaires. The average age of participants was 45.4 years (SD = 12.1); 82.8% were female, 56.6% were white, and 35.6% were African American. Participants had an average weight of 92.3 kg (SD = 14.4) and an average BMI of 32.1 (SD = 3.9).

Participants completed the baseline questionnaire online, following which they were randomized to one of two treatment arms: Web-based tailored (“expert system”) weight management materials or Web-based nontailored (“user navigated”) weight management materials, with the latter serving as the control group. Following the 6-week weight management program, participants were assessed by an Internet-based survey 3, 6, and 12 months after baseline assessment.

Email notices of the availability of each follow-up questionnaire were sent to participants, who received as many as 21 email reminders over a 3-week period before being considered as a nonrespondent to each follow-up assessment. All participants originally enrolled at baseline were sent email prompts to complete the Web-based 6-month and 12-month surveys, regardless of response status at earlier follow-up waves. No incentive was offered for completion of the online follow-up surveys.

Of the participants enrolled at baseline, 31% responded to the Web-based 3-month follow-up survey, while 21% responded to the 6-month survey, and 15% responded to the 12-month survey. There were no significant differences in attrition by baseline assignment to treatment arm, baseline BMI, or a variety of other baseline measures. However, given that 85% of the baseline participants were lost to follow-up at the 12-month measurement, it is important to explore the effect that this may have had on the results of this study. The goal of the present paper is to examine the reasons for loss to follow-up in the online weight management intervention and examine possible differences between those who were lost and those who were retained in the study.

### Design and Implementation of the Nonresponse Follow-Up Survey

Given that only 21% of the original participants responded to the online 6-month survey, a small pilot study was conducted among nonrespondents to explore possible reasons for dropout. A telephone survey was used to contact 104 participants, of whom 44 agreed to be interviewed and provide relevant information, with 42 providing weight information. The results of the 6-month follow-up are reported elsewhere [15]. No significant differences were found between 6-month respondents and the 6-month nonrespondent sample interviewed by telephone in terms of weight loss, weight, motivation to manage weight, self-efficacy, or program rating. Of the 44 interviewed, 20 (45%) reported not receiving any email notification for either the 3- or 6-month follow-up survey. The success of this pilot study led us to design a larger follow-up after the 12-month online survey.

One of the four KP regions participating in the weight management intervention was dropped because of administrative delays in approving the follow-up studies. The Institutional Review Boards at the remaining three KP regions, as well as Group Health Cooperative and the University of Michigan, approved the study protocol. This left us with a total of 3260 baseline participants—1681 treatment and 1579 control participants. The pattern of response to the three online follow-up surveys is shown in Table 1.

**Table 1:** Response pattern to three online follow-up surveys

Response Pattern			Treatment		Control		All	
3-month	6-month	12-month	No.	%	No.	%	No.	%
R	R	R	202	12.0	162	10.3	364	11.2
NR	R	R	21	1.2	21	1.3	42	1.3
R	NR	R	29	1.7	31	2.0	60	1.8
NR	NR	R	17	1.0	16	1.0	33	1.0
R	R	NR	115	6.8	113	7.2	228	7.0
NR	R	NR	34	2.0	29	1.8	63	1.9
R	NR	NR	177	10.5	192	12.2	369	11.3
NR	NR	NR	1086	64.6	1015	64.3	2101	64.4

**Total**			1681	100.0	1579	100.0	3260	100.0

R = respondent; NR = nonrespondent

As shown in Table 1, the majority of participants (64.4% overall) did not complete any of the follow-up surveys, while 499 (15.3%) completed the 12-month follow-up (whether or not they completed earlier follow-up surveys). The pattern of response across the three waves of follow-up is quite similar between the treatment and control groups.

The study reported here follows up on those who did not respond to the 12-month survey, regardless of their response to the 3- and 6-month surveys. This left us with 2761 participants eligible for the nonresponse follow-up study, 1412 from the treatment group and 1349 from the control group. Given that those who did not do any of the follow-up surveys comprised the largest group, we drew a systematic sample of cases from this group, but selected all those who did one or two of the follow-up surveys but not all three. In this way, we selected a total of 700 baseline participants, 350 from the treatment group and 350 from the control group. The sample size was determined largely by budget constraints.

We note that this is not an equal probability selection of nonrespondents. Those who did none of the follow-up surveys are under-represented in this sample relative to those who did one or more follow-ups. However, we used unweighted analyses because our focus was more on the differences between modes (see below) and differences between treatment arms than on the differences by pattern of nonresponse. However, we also conducted weighted analyses, and these led to similar conclusions as those presented here.

The rationale for the design of the 12-month nonresponse follow-up was based on several expectations:

- We hypothesized that much of the attrition may be due to reasons unrelated to a particular arm of the weight management intervention.
- We expected that a change in data collection mode may bring many of the nonrespondents back in.
- We wanted to evaluate the cost-effectiveness of alternative follow-up strategies.

In particular, while telephone follow-up is often an effective way of increasing response to other modes of data collection (especially mail), it is both more costly and raises concerns about social desirability and the effects of instrument design. The presence of an interviewer is known to affect reporting of socially sensitive information [16]. In addition, the visual versus aural presentation of survey questions may affect the answers obtained in the two modes [17]. For these reasons we were interested in the efficacy of a mail follow-up relative to a telephone follow-up. We expected the mail follow-up to be more similar to the Web in the measurement properties and to be cheaper than the telephone, but to take longer and be less effective than the telephone in gaining cooperation from nonrespondents to the online survey.

The use of the telephone for follow up is often predicated on the assumption that the online questionnaire was received, and that those who did not return it may need to be persuaded to participate. Switching from one self-administered mode (Web) to another (mail) is a largely untested approach. But if it works, it has potential cost benefits as well as the advantage of greater comparability of measurement. For this reason, we embedded a mode experiment in the nonresponse follow-up study, with 300 of the nonrespondents being randomly assigned to telephone and 400, to mail. The disproportionate allocation reflects the cost differential between the two modes.

The mail follow-up survey involved a single mailing. Questionnaires were mailed with a KP return address and a cover letter signed by each of the three KP regional directors. Completed questionnaires were returned to Group Health Cooperative (GHC) for processing. Each mailed questionnaire included a US $5 bill as a token of appreciation for completing the questionnaire. The questionnaire was printed on a single 8-1/2” by 14” sheet folded in booklet form and contained a maximum of 13 questions to be answered. Two duplicates were discovered during the mailing process, leaving us with a sample of 398 mail cases.

**Figure 1:**
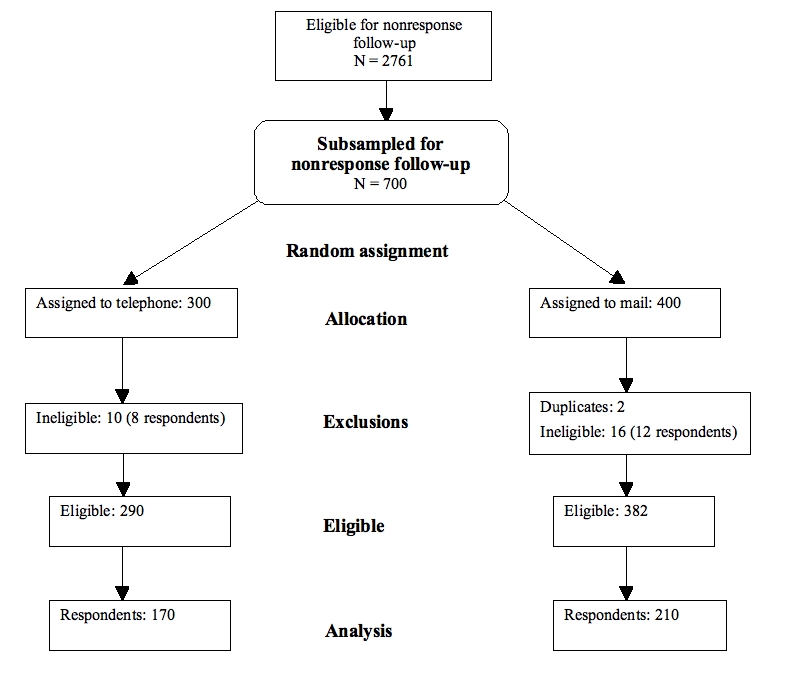
Nonresponse follow-up recruitment flowchart

The telephone survey was conducted by trained interviewers at GHC. No advance letter or incentive was used. Up to 15 call attempts were made on various days and at various times of day. The average interview length was 5.36 minutes. Figure 1 presents a flowchart of the nonresponse follow-up recruitment process, following the CONSORT model.

Data from the mail and telephone survey were combined into a single data file and merged with the baseline and online follow-up responses for analysis. We first examine the results of the nonresponse follow-up survey before looking at correlates of attrition and nonresponse in both the 12-month online survey and the nonresponse follow-up survey.

## Results

### The Nonresponse Follow-Up Survey

#### Response Rates

Based on prior research, we anticipated a 40% response rate and expected the telephone mode to yield a higher response rate than mail. Overall, 400 out of 698 responded to the nonresponse follow-up, for a 57.3% response rate. Unfortunately, we learned after the fact that 26 cases in our sample (including 20 respondents) were not eligible for the intervention, so the final count was 380 respondents. Subsequent analyses are based on these 380 respondents.

Of the 290 cases in the telephone sample, 170 responded, for a 58.6% response rate; for those in mail sample, 210 out of 382 responded, for a 55.0% response rate. These rates did not differ significantly (*χ* ^2^_1_= 0.9, *P* = .34). However, the mail survey took substantially longer to complete, and, if time were a factor, the telephone mode would yield a higher response rate within 2 weeks, or even a month, after the start of follow-up. The median response time for the mail survey was 10 days (mean = 16.2, SD = 13.9), while that for the telephone survey was 8 days (mean = 8.9, SD = 6.5). If we had cut off data collection at 14 days, we would have obtained 61.9% of the mail respondents and 78.8% of the telephone respondents. Similarly, cutting off data collection at 28 days would acquire 85.7% of mail respondents and all but one of the telephone respondents. The last completed mail questionnaire was received some 6 months after the initial mailing.

Overall, the number of online follow-up surveys that participants completed is predictive of whether they completed the nonresponse follow-up survey: 50.1% of those who did not respond to any of the three online surveys completed the nonresponse survey, compared with 59.8% of those who did not respond to 2 of the 3 online surveys, and 65.3% of those who did not respond to only 1 of the 3 online surveys (*χ* ^2^_2_= 9.0, *P* = .01). This pattern is similar for both the mail and telephone samples. But, even so, about half of those who did not complete any measurements following baseline were still brought back into the study some 12 months later with the nonresponse follow-up study.

#### Costs of Follow-Up

What are the relative costs of conducting mail versus telephone follow-up surveys of nonrespondents? To produce crude estimates, we took the total cost of each operation and divided by the sample size and the number of completes, respectively. Costs for the telephone included the development of the short computer-assisted telephone interviewing (CATI) instrument and conduct of the survey. Costs for the mail survey included the cost of printing the questionnaires and assembling the mailing packets, the cost of incentives and postage, and the cost of keying the data from the returned questionnaires.

Based on these rough numbers, we estimate that the telephone survey cost about US $34 per sampled case, compared to US $15 for the mail survey. On the basis of completed questionnaires, the relative costs were approximately US $57 per completed case for the telephone survey and US $28 for the mail survey. Given this cost differential, the mail survey was not only almost as effective in terms of response rate as the telephone survey, but also substantially less expensive.

#### Reasons for Nonresponse

We included a few questions in the nonresponse follow-up survey to ascertain reasons for earlier nonresponse. Based on the results of the 6-month pilot follow-up, these questions primarily focused on technical barriers to completing the online surveys.

We first asked whether participants recalled receiving the email message for the 12-month online follow-up survey. If they had, we asked if they recalled reading the message. If they did so, we went on to ask if they clicked on the link to access the survey. For those who did not recall receiving an email message, we asked them to provide their current email address. The results from this series of questions are presented in Table 2.

**Table 2:** Responses to nonresponse follow-up questions about the email invitations

	Mail (n=210)	Telephone (n=170)	Total (n=380)
Recall receiving email message	53.8% (113/210)	43.5% (74/170)	49.2% (187/380)
Recall reading email message	44.3% (93/210)	28.8% (49/170)	37.4% (142/380)
**Among those not recalling receiving the email message:**			
Do you still have access to email? (% yes)	87.6% (85/97)	96.9% (93/96)	92.2% (178/193)
**Among those not recalling reading the email message:**			
How often do you typically check email? (% at least 1-2 times per week)	73.5% (86/117)	71.9% (87/121)	72.7% (173/238)
How often do you delete email without opening? (% at least sometimes)	65.0% (76/117)	65.3% (79/121)	65.1% (155/238)
**Among those recalling receiving the email message:**			
Did you click on the link to access the survey? (% yes)	47.8% (54/113)	31.1% (23/74)	41.2% (77/187)

Mail respondents reported higher rates of recall for both receiving and reading the email message than did telephone respondents. One reason may be that the mail mode gives respondents more time to consider the question and retrieve information from memory, or even check their email inbox.

An additional 13.8% (36.4% of mail respondents and 16.7% of phone respondents) who answered “no” to the question on recalling receiving the email message still answered that they clicked on the link to the survey. Thus, 26.1% of respondents overall (29.5% of mail and 21.8% of phone) reported clicking on the link for the survey, regardless of whether they recalled receiving or reading the email message.

All respondents to the nonresponse follow-up who said they still had access to email, whether or not they recalled receiving or reading the email invitations, were asked in an open-ended question the main reason for not accessing the website or completing the survey. The responses were classified into several themes, summarized in Table 3. The responses did not appear to differ by mode, so the combined distribution of responses is presented.

  

**Table 3:** Classification of reasons for noncompletion of the online survey into major themes

Theme	No.	%
		
No response to question	21	5.5
**Technical problems**		
Problems accessing or submitting the survey	59	15.5
Email or computer problems (including those with no access to email)	35	9.2
Did not receive or remember message, or treated it as SPAM	103	27.1
**Problems with intervention or survey**		
Lack of interest in or lack of effectiveness of intervention	67	17.6
Survey was boring or too long	8	2.1
Medical or personal reasons	8	2.1
Refused to do survey	8	2.1
No time or bad timing	53	13.9
**Other**		
Don’t remember	5	1.3
Did complete the survey	13	3.4
		
**Total**	380	100.0

By far the largest number of nonresponse follow-up respondents (103) gave reasons related to not recalling receipt of the email message. If we treat this as a technical problem, we see that a total of 197 respondents (51.8%) gave reasons related to technical issues or email as a means of communication. In contrast, 144 (37.9%) gave reasons related to the intervention or the survey itself (see Table 3). This suggests that the medium of communication (email) may be as much if not more of a factor in attrition as the intervention. This is one of the reasons we believe that the mail and telephone follow-up surveys were successful. Of course, these are post hoc justifications of why participants did not do the online survey, and we don’t know the reasons for noncompletion for those participants who did not respond to the nonresponse follow-up survey.

To explore whether some types of participants may be more likely to report technical problems than others, we collapsed the reasons in Table 3 into three broad groups (technical, substantive, and other) and ran a multinomial logistic regression using baseline demographic and design variables as predictors. Only age was significantly (*P* = .03) associated with the type of reason given, with older people more likely to give technical reasons for noncompletion of the 12-month online survey: 43% of those 45 years and younger cited technical problems, compared to 55% of those 46-60 and 67% of those 61 or older.

#### Differential Response by Mode

While we found no significant differences in the overall response rate to the nonresponse follow-up by mode, are some people more likely to respond to one follow-up mode than the other? While the response rates are similar, it could be that the mail follow-up brings in different types of people than the telephone follow-up survey. To examine this, we ran a logistic regression model predicting whether a case was a mail or telephone respondent, conditional on having completed the follow-up survey. None of the demographic or design variables we examined—age, gender, race, education, KP region, or treatment group—was statistically significant. This suggests that the differences in the answers to the survey questions by mode (see below) are likely due to the features of the modes themselves rather than differential selection into the two groups.

#### Mode Effects

Several rating scales were included in the follow-up questionnaires. Based on the literature (eg, [18]), we expected two types of response effects. First, response options presented early in a list are more likely to be selected in visual presentation modes (mail and Web), while those presented later in a list are more likely to be selected in aural modes (telephone). In others words, primacy effects are likely in the mail mode, while recency effects are more likely in the telephone mode [19]. The second effect is that of socially desirable responding, with more positive (ie, socially desirable) responses expected on the telephone given the presence of an interviewer.

Four of the ratings were presented with the most negative response (eg, not at all confident, not at all satisfied, not at all motivated) listed first. The responses were read in the same order on the telephone. The response order effect (primacy in mail, recency in telephone) is expected to produce lower means (more negative) for the mail respondents, as would the social desirability effect. In other words, the two effects are expected to reinforce each other. The means and standard deviations for these items are presented in Table 4. For three of the four items, we find significantly lower means for mail than telephone: confidence in managing one’s own weight (*t*_341_ = 2.64, *P* = .009), confidence in maintaining recommended levels of physical activity (*t*_352_ = 3.54, *P* < .001), and motivation to manage one’s own weight (*t*_371_ = 2.66, *P* = .008). For the fourth item of this type, a rating of satisfaction with care at KP, no differences were found (*t*_366_ = 0.34, *P* = .73).

One item, a rating of the online weight management information, was ordered from the most positive (excellent) to the least positive response (poor). Here, the response order and social desirability effects are expected to cancel each other out. First, nonresponse to this item was higher than all other items (20% of mail respondents and 25% of phone respondents did not answer), perhaps reflecting the fact that some participants may not have spent much time with the online materials. This is borne out when we look at the treatment group only: of those who did not look at any of the online newsletters, 27.7% did not answer this question, compared to 8.1% for those who looked at one or more newsletters. Among those who did answer the question, we find no significant differences in the mean ratings on this item by mode (*t*_264_ = 0.33, *P* = .74).

**Table 4:** Mean responses (SD) to nonresponse follow-up measures, by mode

Question	Mail	Telephone
Q4. Overall, how would you rate the online weight management information that you received? (1 = Excellent to 5 = Poor)	2.97 (1.02)	2.93 (1.08)
Q5. Currently, how confident are you that you can manage your weight? (1 = Not at all to 5 = Extremely)	2.05 (0.97)	2.34^*^ (1.09)
Q6. Using a scale from 0 to 10, where 0 means not at all motivated and 10 means extremely motivated, how motivated are you to manage your weight?	6.05 (2.57)	6.72^*^ (2.35)
Q7. How confident are you that you can maintain recommended levels of physical activity? (1 = Not at all to 5 = Extremely)	2.36 (1.09)	2.76^*^ (1.14)
Q8. How much do you currently weigh? (pounds)	209.6 (46.61)	208.6 (46.06)
Q9. Finally, how satisfied are you overall with your care at Kaiser Permanente? (1 = Not at all to 5 = Extremely)	3.60 (1.00)	3.56 (0.94)

^*^ *P* < .05, comparing mail versus telephone (t-test, see text for P-values)

Nonresponse to the most critical question (“How much do you currently weigh?”) did not differ by mode, with 8.1% of mail and 8.8% of telephone respondents not providing an answer to this question. Among those who did answer, the telephone respondents reported a lower weight on average than the mail respondents; however, this did not reach statistical significance (*t*_332_ = 0.19, *P* = .85; see Table 4). If we examine reported weight loss from baseline to the 12-month nonresponse follow-up survey (the key dependent variable), we find significant effects, with the telephone respondents reporting greater weight loss than the mail respondents, whether reported in kilograms (an average weight loss of 3.30 kg for telephone respondents and 1.19 kg for mail respondents; *t*_320_ = 3.1, *P* = .002) or BMI (an average BMI reduction of 1.20 for telephone respondents and 0.45 for mail respondents; *t*_315_= 2.96, *P* = .003). Given the known social desirability effects associated with the telephone, we believe that the mail responses are more “honest” than those provided over the telephone. This is consistent with the view in the mode effects literature (eg, [17,18]) that higher reports of socially undesirable behaviors or attributes (and lower reports of socially desirable ones) reflect greater accuracy of reporting. Our findings suggest that if we had done a telephone follow-up only, we would have overestimated the weight loss for both the treatment and control groups (the mode difference does not interact with experimental condition). Given that the mail mode is more similar to the online measurement used for both baseline and follow-up surveys, we believe that the smaller weight loss estimated for the mail respondents more closely reflects the truth.

The evidence for social desirability bias in the telephone responses echoes findings from other studies. For example, Eicheldinger et al [20] conducted a follow-up of nonrespondents to the Consumer Assessment of Health Plans Study (CAHPS), randomly assigning participants to telephone or mail (using overnight delivery) follow-up. While their response rates were lower than ours (23.7% for mail and 27.1% for phone), they found that those who responded by telephone were more likely to report the most positive response to 13 of the 20 performance measures. Similarly, in a study of employees at a large company who were randomly assigned to Web or telephone modes of data collection [21], significant differences were obtained for mean satisfaction with the health insurance plan (6.88 for Web and 7.32 for telephone, *P* < .05) and for mean self-rated health (3.51 for Web and 3.79 for telephone, *P* < .01). Similar effects are found in comparisons of mail versus telephone [22] and Web versus telephone [23]. However, our results suggest that it may not just be social desirability effects at work; differences in format or layout of the items may also produce mode effects [24].

### Modeling Nonresponse

In addition to data from the nonresponse follow-up survey, we also have information on all participants from the baseline survey. In this section, we use these data to examine correlates of nonresponse to the 12-month online survey and also to the mail and telephone nonresponse follow-up surveys.

In contrast to cross-sectional sample surveys in which little is known about sample members who do not participate, one of the advantages of an (online) intervention such as this is that a lot of information may be collected at baseline, and these data can be used to examine who drops out and who does not, among those who enrolled. The data can be used not only to examine patterns of differential attrition among subgroups, but also to statistically adjust for such patterns at the time of analysis.

We ran several logistic regression models, first predicting response or nonresponse to the 12-month online survey, then, predicting response or nonresponse to the follow-up survey (among those included in the eligible for nonresponse follow-up sample). We briefly summarize these models below.

#### Response to the 12-Month Online Survey

The first model included the following baseline demographic and design variables: age, gender, race, education, KP region, BMI, study assignment (treatment or control). The coefficient of determination, *R* ^2^, [25] for this model was 0.030, while the Nagelkerke [26] max-rescaled *R* ^2^ measure was 0.052 (see [27] for a discussion of alternative pseudo *R* ^2^ indices). This suggests that these baseline variables do not do a very good job of predicting whether a participant will be a respondent or nonrespondent to the 12-month online survey. This is reassuring in that the attrition does not appear to vary much by these baseline characteristics. Specifically, the dropout rates for the treatment and control groups did not differ significantly. There were no significant differences in 12-month completion by baseline BMI or by gender. The KP regions differed significantly in their 12-month completion rates, ranging from 10.2% to 18.8% across the three regions in the study, but there was no significant interaction with treatment group. Age was associated with a significant (*P* = .01) positive effect on completion. Minorities (African Americans and those of other races) were significantly (*P* = .008) less likely to complete the 12-month survey, as were those with lower levels of education (*P* = .009). Both of these variables are correlated with lower levels of Internet access and may be associated with greater risk of losing such access over the life of the study [28,29].

To this model, we added a set of behavioral and attitudinal measures related to the online intervention from the baseline survey. These included whether the participant had received medical advice to lose weight, how successful they were at losing weight in the past, their weight loss goals for the program, their motivation for losing weight, frequency of exercise and physical activity, self-rated health, and satisfaction with KP. The addition of these variables did not significantly improve the fit of the model (*χ* ^2^_14_ = 30.7 [for ∆ in −2 LogL]), producing a max-rescaled *R* ^2^ of 0.068. Among the added variables, only level of physical activity (*P* = .03), with those doing light exercise being more likely to complete the survey than those doing moderate to heavy activity, and self-rated health (*P* = .009), with better health associated with higher rates of completion, were statistically significant under the model. In other words, baseline measures of motivation to lose weight, weight loss target, satisfaction with KP, assignment to treatment or control group, and the like, were not significantly associated with completion of the 12-month online survey. This provides some reassurance that nonresponse bias may not be large—at least in terms of variables measured at baseline.

A final model added a set of process measures from the intervention, namely whether the participant completed the 3- and 6-month online surveys. As expected, nonresponse to one of the early follow-up surveys was highly predictive of nonresponse to the 12-month follow-up survey, with conditional odds ratios of 4.0 (95% CI, 2.90-5.53) and 13.1 (95% CI, 9.78-17.62) for completion of the 3- and 6-month survey, respectively. In addition, those in the treatment group were given access to three online newsletters as part of the intervention. Among this group, the number of newsletters they accessed on the website was predictive of 12-month survey completion. The odds ratio of being a respondent at 12 months for those who opened no newsletters was 0.14 (95% CI, 0.07-0.26) relative to those who accessed all three, while for those who accessed one newsletter, it was 0.49 (95% CI, 0.29-0.83), and for those who accessed two newsletters, it was 0.52 (95% CI, 0.32-0.86). These limited process indicators suggest two conclusions: (1) those who are actively engaged in the intervention (ie, who show evidence of visiting the website and accessing material) have higher completion rates, and (2) those who responded to earlier follow-up surveys are more likely to respond to the final (12-month) follow-up survey.

These conclusions, in turn, have two implications. First, online interventions can provide researchers with a wide variety of measures of active engagement in the program [30]. These indicators can include number of sessions logged in, time spent online, number of pages viewed, and so on. Such process data or paradata [31] can be routinely captured as part of such online interventions and can be useful not only for understanding how much time and attention is spent on different parts of the website (with a view to identifying areas for improvement), but also as a measure of how much participants are being exposed to the stimulus material. This could serve as an important mediator variable in analyses of various outcome measures. Second, when multiple follow-ups are part of the design, nonresponse to earlier follow-up surveys can identify participants at risk for dropout, permitting researchers to target intervention strategies aimed at retaining such participants in the study. The responsive design strategies being developed to reduce nonresponse in surveys (eg, [32]) can similarly be deployed to counter nonresponse in online interventions. Online studies not only permit targeted or tailored interventions, but also tailoring of data collection and follow-up strategies.

#### Response to the Nonresponse Follow-Up Survey

The second set of models parallels the first, but focuses on completion of the mail or telephone follow-up survey, among those included in the nonresponse follow-up study. These models are based on 672 eligible participants included in the nonresponse follow-up, 380 of whom completed either the mail or telephone survey. The max-rescaled *R* ^2^ measure for the demographic and design variables was 0.067. Only age remained a significant (*P* < .001) predictor of response to the nonresponse follow-up survey, with older people more likely to complete the survey. Interestingly, while African Americans were less likely than whites to complete the 12-month online survey, they appeared slightly but not significantly (*P* = .36) more likely (OR = 1.25, 95% CI, 0.85-1.85) to complete the mail or telephone follow-up survey. Similar effects are found for those with high school or lower education relative to those with a college degree (*P* = .59, OR = 1.3, 95% CI, 0.75-2.26). This may provide some support for the observation that those at greatest risk of losing access to the Internet (older persons, minorities, those with lower education) may be brought back into the analytic sample with alternative modes of data collection.

As before, adding the baseline behavioral and attitudinal measures to this model did not improve the fit, relative to the base model. Only one of the added predictors was statistically significant (*P* < .05), with those who spend 2 hours or more a day in front of the TV or computer outside of work being less likely to respond to the follow-up survey (*P* = .02). This suggests that the decision to complete the nonresponse follow-up survey is made largely independent of the original decisions regarding participation in the online intervention.

#### Adjusting for Nonresponse

One of the goals of conducting the nonresponse follow-up survey among a sample of nonrespondents, in addition to exploring reasons for nonresponse, was to obtain data to inform statistical adjustment for nonresponse. In his review of the attrition problem, Eysenbach [2] argues that an intent-to-treat analysis, in which all dropouts are assumed to have negative or neutral outcomes, is the only way to avoid selection bias. We argue rather that weighting or imputation based on informed models of attrition or dropout requires fewer assumptions about the missing cases. As Hollis and Campbell [33, p. 673-674] note, “…no imputation method can give an unbiased estimate of the treatment effect unless the assumptions made about the missing data are valid.” A nonresponse follow-up study allows one not only to reduce the amount of missing data, but also to evaluate the missing data assumptions. To quote Hollis and Campbell [33, p. 674] again, “To fully appreciate the potential influence of missing responses, some form of sensitivity analysis is recommended, examining the effect of different strategies on the conclusions.”

In work described elsewhere [34], we used the data from the nonresponse follow-up to multiply-impute data for the remaining 12-month online nonrespondents [35]. This method utilizes all available data, while accounting for the uncertainty due to imputation. Using a complete case analysis from the 12-month online respondents only, we would reach a conclusion that the treatment had a statistically significant effect on weight loss at 12 months relative to the control. However, using the data from the nonresponse follow-up to impute the missing 12-month cases, we would conclude that the differences between treatment and control, although still in the expected direction, do not reach statistical significance. These models are limited by the small number of cases included in the nonresponse follow-up relative to the number of nonrespondent cases and by the differences we found between the two modes of follow-up. Therefore, these results can only be viewed as suggestive. However, they allow us to explore the sensitivity of the substantive models to different assumptions about the missing data at the 12-month follow-up.

## Discussion

Our study has several potential limitations. First, the nonresponse follow-up was conducted within the context of a weight management intervention, which was restricted to overweight and obese members of a health maintenance organization (HMO) with regular Internet access. This may limit generalizability to other populations and settings. Second, this study did not test different ways to enhance the response rate to the 12-month online survey (eg, by using incentives). The success of the follow-up effort may be conditional on the initial response obtained. Third, this was a small-scale exploratory study embedded in a larger study. The small sample size may limit our ability to draw statistically reliable conclusions.

Nonetheless, we have learned a number of things from this exploratory study. First, a significant proportion of those who drop out of an online RCT or intervention can be brought back by switching modes of data collection. A variety of technical reasons, unrelated to the online intervention, can account for a substantial proportion of such dropout, and modes switches are an effective counter to the uncertainties of using email as a communication medium.

Second, we learned that mail is almost as effective as the telephone for such follow-up. Further, it is significantly cheaper, and it is more similar to the original online mode in terms of visual design and response styles and shares the absence of social desirability effects associated with interviewers. For these reasons, we believe that the mail survey produced responses that are more comparable to the online responses than did the telephone survey. Telephone calls can be a useful tool for prompting or reminding respondents to return their questionnaires, but we believe that mail is a cost-effective method of following up online nonrespondents, if time is not a critical factor. On the basis of this work, we implemented a mail-only nonresponse follow-up study in a second controlled trial of a weight loss program [30].

Third, nonresponse follow-up studies such as this not only increase the number of cases for analysis but also help us understand the differences between those who drop out and those who complete all follow-up surveys. In other words, our analyses of treatment effects are not forced to rely on the often-heroic assumptions required by complete-case analysis. Nonresponse, or attrition, bias can be reduced in two ways: one is to reduce the rate of attrition, and the other is to reduce or measure the differences between those who drop out and those who don’t [36]. We believe that following up nonrespondents—whether a sample of them as we did here, or all nonrespondents—using a different mode is a cost-effective way of increasing the analytic power and reducing the potential bias that may result from the relatively high rates of dropout experienced in online interventions and follow-up surveys.

## Abbreviations

BMI: body mass index
GHC: Group Health Cooperative
KP: Kaiser Permanente
RCT: randomized controlled trial
